# Psilocybin produces substantial and sustained decreases in depression and anxiety in patients with life-threatening cancer: A randomized double-blind trial

**DOI:** 10.1177/0269881116675513

**Published:** 2016-11-30

**Authors:** Roland R Griffiths, Matthew W Johnson, Michael A Carducci, Annie Umbricht, William A Richards, Brian D Richards, Mary P Cosimano, Margaret A Klinedinst

**Affiliations:** 1 Department of Psychiatry and Behavioral Sciences, Johns Hopkins University School of Medicine, Baltimore, MD, USA; 2 Department of Neuroscience, Johns Hopkins University School of Medicine, Baltimore, MD, USA; 3 Sidney Kimmel Comprehensive Cancer Center, Johns Hopkins University School of Medicine, Baltimore, MD, USA

**Keywords:** Psilocybin, hallucinogen, cancer, anxiety, depression, symptom remission, mystical experience

## Abstract

Cancer patients often develop chronic, clinically significant symptoms of depression and anxiety. Previous studies suggest that psilocybin may decrease depression and anxiety in cancer patients. The effects of psilocybin were studied in 51 cancer patients with life-threatening diagnoses and symptoms of depression and/or anxiety. This randomized, double-blind, cross-over trial investigated the effects of a very low (placebo-like) dose (1 or 3 mg/70 kg) vs. a high dose (22 or 30 mg/70 kg) of psilocybin administered in counterbalanced sequence with 5 weeks between sessions and a 6-month follow-up. Instructions to participants and staff minimized expectancy effects. Participants, staff, and community observers rated participant moods, attitudes, and behaviors throughout the study. High-dose psilocybin produced large decreases in clinician- and self-rated measures of depressed mood and anxiety, along with increases in quality of life, life meaning, and optimism, and decreases in death anxiety. At 6-month follow-up, these changes were sustained, with about 80% of participants continuing to show clinically significant decreases in depressed mood and anxiety. Participants attributed improvements in attitudes about life/self, mood, relationships, and spirituality to the high-dose experience, with >80% endorsing moderately or greater increased well-being/life satisfaction. Community observer ratings showed corresponding changes. Mystical-type psilocybin experience on session day mediated the effect of psilocybin dose on therapeutic outcomes.

**Trial Registration**

ClinicalTrials.gov identifier: NCT00465595

## Introduction

Cancer patients often develop a chronic, clinically significant syndrome of psychosocial distress having depressed mood, anxiety, and reduced quality of life as core features, with up to 40% of cancer patients meeting criteria for a mood disorder (Holland et al., 2013; Mitchell et al., 2011). In cancer patients, depression and anxiety have been associated with decreased treatment adherence (Arrieta et al., 2013; Colleoni et al., 2000), prolonged hospitalization (Prieto et al., 2002), decreased quality of life (Arrieta et al., 2013; Skarstein et al., 2000), and increased suicidality (Shim and Park, 2012). Depression is an independent risk factor of early death in cancer patients (Arrieta et al., 2013; Pinquart and Duberstein, 2010). Antidepressants and, less frequently, benzodiazepines are used to treat depressed mood and anxiety in cancer patients, although evidence suggesting efficacy is limited and conflicting, and benzodiazepines are generally only recommended for short-term use because of side effects and withdrawal (Grassi et al., 2014; Ostuzzi et al., 2015; Walker et al., 2014). Although psychological approaches have shown only small to medium effects in treating emotional distress and quality of life, with low quality of reporting in many trials (Faller et al., 2013), there are several promising interventions utilizing existential orientations to psychotherapy (Breitbart et al., 2015; Spiegel, 2015).

The classic hallucinogens, which include psilocybin (psilocin) and (+)-lysergic acid diethylamide (LSD), are a structurally diverse group of compounds that are 5-HT_2A_ receptor agonists and produce a unique profile of changes in thoughts, perceptions, and emotions (Halberstadt, 2015; Nichols, 2016). Several unblinded studies in the 1960s and 70s suggested that such compounds might be effective in treating psychological distress in cancer patients (Grof et al., 1973; Kast, 1967; Richards et al., 1977); however, these studies did not include the comparison conditions that would be expected of modern psychopharmacology trials.

Subsequently, human research with these compounds was halted for almost three decades because of safety and other concerns raised in response to widespread non-medical use in the 1960s. Recent resumption of clinical research with these compounds has established conditions for safe administration (Johnson et al., 2008; Studerus et al., 2011).

Two recent double-blind, placebo-controlled studies with the classic hallucinogens psilocybin (Grob et al., 2011) and LSD (Gasser et al., 2014) examined effects in 12 patients with life-threatening illness, including cancer. Both studies showed promising trends toward decreased psychological distress. Of most relevance to the present study with psilocybin, Grob and colleagues showed that a low-moderate dose of psilocybin (14 mg/70 kg) decreased a measure of trait anxiety at 1 and 3 months and depressed mood at 6-month follow-up. Also relevant, a recent open-label pilot study in 12 patients with treatment-resistant depression showed marked reductions in depressive symptoms 1 week and 3 months after administration of 10 and 25 mg of psilocybin in two sessions separated by 7 days (Carhart-Harris et al., 2016).

The present study provides the most rigorous evaluation to date of the efficacy of a classic hallucinogen for treatment of depressed mood and anxiety in psychologically distressed cancer patients. The study evaluated a range of clinically relevant measures using a double-blind cross-over design to compare a very low psilocybin dose (intended as a placebo) to a moderately high psilocybin dose in 51 patients under conditions that minimized expectancy effects.

## Methods

### Study participants

**Figure 1.**
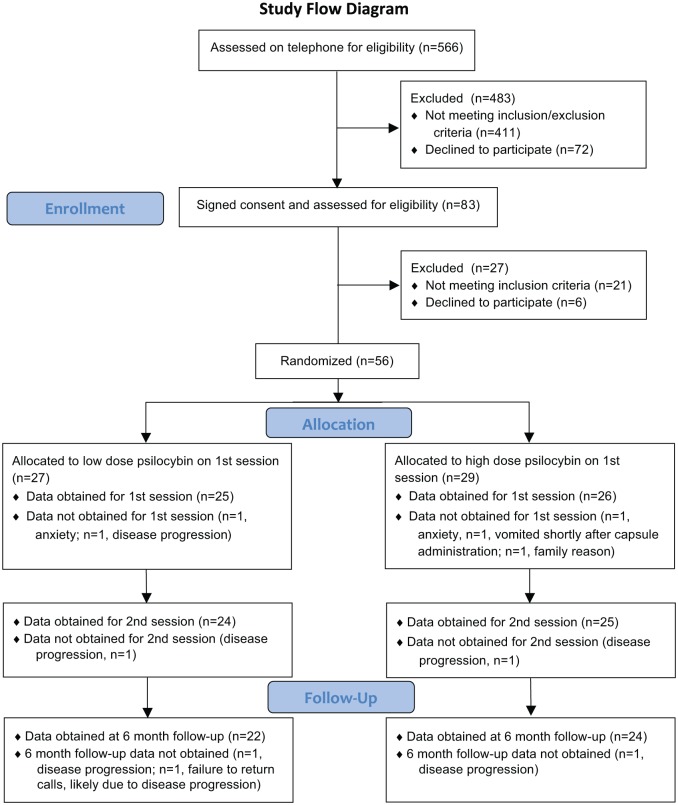
Flow diagram showing participation across the study.

Participants with a potentially life-threatening cancer diagnosis and a DSM-IV diagnosis that included anxiety and/or mood symptoms were recruited through flyers, internet, and physician referral. Of 566 individuals who were screened by telephone, 56 were randomized. Figure 1 shows a CONSORT flow diagram. Table 1 shows demographics for the 51 participants who completed at least one session. The two randomized groups did not significantly differ demographically. All 51 participants had a potentially life-threatening cancer diagnosis, with 65% having recurrent or metastatic disease. Types of cancer included breast (13 participants), upper aerodigestive (7), gastrointestinal (4), genitourinary (18), hematologic malignancies (8), other (1). All had a DSM-IV diagnosis: chronic adjustment disorder with anxiety (11 participants), chronic adjustment disorder with mixed anxiety and depressed mood (11), dysthymic disorder (5), generalized anxiety disorder (GAD) (5), major depressive disorder (MDD) (14), or a dual diagnosis of GAD and MDD (4), or GAD and dysthymic disorder (1). Detailed inclusion/exclusion criteria are in the online Supplementary material. The Johns Hopkins IRB approved the study. Written informed consent was obtained from participants.

**Table 1.** Participant demographics for all participants and for both of the dose sequence groups separately^+^.

Measure	Low-Dose-1st (High-Dose-2nd) (*n*=25)	High-Dose-1st (Low-Dose-2nd) (*n*=26)	All Participants (*n*=51)
Gender (% female)	48%	50%	49%
Age in years (mean, SEM)	56.1 (2.3)	56.5 (1.8)	56.3 (1.4)
Race/Ethnicity			
White	92%	96%	94%
Black/African American	4%	4%	4%
Asian	4%	0%	2%
Education			
High school	4%	0%	2%
College	32%	58%	45%
Post-graduate	64%	42%	53%
Relationship status (married or living with partner)	72%	65%	69%
Lifetime use of hallucinogens			
Percent reporting any past use	56%	36%	45%
Years since last use (mean, SEM)	30.9 (3.2)	30.0 (4.5)	30.6 (2.6)
Recent use of cannabis or dronabiol			
Percent reporting recent use	52%	42%	47%
Users use per month (mean, SEM)	4.7 (1.6)	7.0 (2.1)	5.8 (1.3)
Cancer prognosis at time of enrollment			
Possibility of recurrence	32%	38%	35%
Recurrent/metastatic (>2yr anticipated survival)	32%	42%	37%
Recurrent/metastatic (<2yr anticipated survival)	36%	19%	27%
Psychiatric symptoms^a^			
Depressed mood	72%	65%	69%
Anxiety	68%	58%	63%
Prior use of medication for anxiety or depression^b^	52%	50%	51%

+ There were no significant differences between the two dose sequence groups on any demographic variable (*t*-tests and chi-square tests with continuous and categorical variables, respectively).

a Psychiatric symptom classification was based on SCID (DSM-IV) diagnoses. All had a DSM-IV diagnosis: chronic adjustment disorder with anxiety (11 participants), chronic adjustment disorder with mixed anxiety and depressed mood (11), dysthymic disorder (5), generalized anxiety disorder (GAD) (5), major depressive disorder (MDD) (14), or a duel diagnosis of GAD and MDD (4), or GAD and dysthymic disorder (1). Depressed mood was defined as meeting criteria for MDD, dysthymic disorder, or adjustment disorder with anxiety and depressed mood, chronic. Anxiety was defined as meeting criteria for GAD, adjustment disorder with anxiety, chronic, or adjustment disorder with anxiety and depressed mood, chronic.

b Data in this row refer to percentage of participants who had received antidepressant or anxiolytic medication after the cancer diagnosis but had terminated the medication sometime before study enrollment because they had found it to be unsatisfactory.

### Study design and overview

A two-session, double-blind cross-over design compared the effects of a low versus high psilocybin dose on measures of depressed mood, anxiety, and quality of life, as well as measures of short-term and enduring changes in attitudes and behavior. Participants were randomly assigned to one of two groups. The Low-Dose-1st Group received the low dose of psilocybin on the first session and the high dose on the second session, whereas the High-Dose-1st Group received the high dose on the first session and the low dose on the second session. The duration of each participant’s participation was approximately 9 months (mean 275 days). Psilocybin session 1 occurred, on average, approximately 1 month after study enrollment (mean 28 days), with session 2 occurring approximately 5 weeks later (mean 38 days). Data assessments occurred: (1) immediately after study enrollment (Baseline assessment); (2) on both session days (during and at the end of the session); (3) approximately 5 weeks (mean 37 days) after each session (Post-session 1 and Post-session 2 assessments); (4) approximately 6 months (mean 211 days) after Session 2 (6-month follow-up).

### Interventions

#### Meetings with session monitors

After study enrollment and assessment of baseline measures, and before the first psilocybin session, each participant met with the two session monitors (staff who would be present during session days) on two or more occasions (mean of 3.0 occasions for a mean total of 7.9 hours). The day after each psilocybin session participants met with the session monitors (mean 1.2 hours). Participants met with monitors on two or more occasions between the first and second psilocybin session (mean of 2.7 occasions for a mean total of 3.4 hours) and on two or more occasions between the second session and 6-month follow-up (mean of 2.5 occasions for a mean total of 2.4 hours). Preparation meetings, the first meeting following each session, and the last meeting before the second session were always in person. For the 37 participants (73%) who did not reside within commuting distance of the research facility, 49% of the Post-session 1 meetings with monitors occurred via telephone or video calls.

A description of session monitor roles and the content and rationale for meetings between participants and monitors is provided elsewhere (Johnson et al., 2008). Briefly, preparation meetings before the first session, which included discussion of meaningful aspects of the participant’s life, served to establish rapport and prepare the participant for the psilocybin sessions. During sessions, monitors were nondirective and supportive, and they encouraged participants to “trust, let go and be open” to the experience. Meetings after sessions generally focused on novel thoughts and feelings that arose during sessions. Session monitors were study staff originally trained by William Richards PhD, a clinical psychologist with extensive experience conducting studies with classic hallucinogens. Monitor education varied from college graduate to PhD. Formal clinical training varied from none to clinical psychologist. Monitors were selected as having significant human relations skills and self-described experience with altered states of consciousness induced by means such as meditation, yogic breathing, or relaxation techniques.

#### Psilocybin sessions

Drug sessions were conducted in an aesthetic living-room-like environment with two monitors present. Participants were instructed to consume a low-fat breakfast before coming to the research unit. A urine sample was taken to verify abstinence from common drugs of abuse (cocaine, benzodiazepines, and opioids including methadone). Participants who reported use of cannabis or dronabinol were instructed not to use for at least 24 h before sessions. Psilocybin doses were administered in identically appearing opaque, size 0 gelatin capsules, with lactose as the inactive capsule filler. For most of the time during the session, participants were encouraged to lie down on the couch, use an eye mask to block external visual distraction, and use headphones through which a music program was played. The same music program was played for all participants in both sessions. Participants were encouraged to focus their attention on their inner experiences throughout the session. Thus, there was no explicit instruction for participants to focus on their attitudes, ideas, or emotions related to their cancer. A more detailed description of the study room and procedures followed on session days is provided elsewhere (Griffiths et al., 2006; Johnson et al., 2008).

#### Instructions to participants and monitors to facilitate dose condition blinding and minimize expectancy effects

Expectancies, on part of both participants and monitors, are believed to play a large role in the qualitative effects of psilocybin-like drugs (Griffiths et al., 2006; Metzner et al., 1965). Although double-blind methods are usually used to protect against such effects, expectancy is likely to be significantly operative in a standard drug versus placebo design when the drug being evaluated produces highly discriminable effects and participants and staff know the specific drug conditions to be tested. For these reasons, in the present study a low dose of psilocybin was compared with a high dose of psilocybin, and participants and monitors were given instructions that obscured the actual dose conditions to be tested. Specifically, they were told that psilocybin would be administered in both sessions, the psilocybin doses administered in the two sessions might range anywhere from very low to high, the doses in the two sessions might or might not be the same, sensitivity to psilocybin dose varies widely across individuals, and that at least one dose would be moderate to high. Participants and monitors were further strongly encouraged to try to attain maximal therapeutic and personal benefit from each session.

#### Dose conditions

The study compared a high psilocybin dose (22 or 30 mg/70 kg) with a low dose (1 or 3 mg/70 kg) administered in identically appearing capsules. When this study was designed, we had little past experience with a range of psilocybin doses. We decreased the high dose from 30 to 22 mg/70 kg after two of the first three participants who received a high dose of 30 mg/70 kg were discontinued from the study (one from vomiting shortly after capsule administration and one for personal reasons). Related to this decision, preliminary data from a dose-effect study in healthy participants suggested that rates of psychologically challenging experiences were substantially greater at 30 than at 20 mg/70 kg (Griffiths et al., 2011). The low dose of psilocybin was decreased from 3 to 1 mg/70 kg after 12 participants because data from the same dose-effect study showed significant psilocybin effects at 5 mg/70 kg, which raised concern that 3 mg/70 kg might not serve as an inactive placebo.

### Outcome measures

#### Cardiovascular measures and monitor ratings assessed throughout the session

Ten minutes before and 30, 60, 90, 120, 180, 240, 300, and 360 min after capsule administration, blood pressure, heart rate, and monitor ratings were obtained as described previously (Griffiths et al., 2006). The two session monitors completed the Monitor Rating Questionnaire, which involved rating or scoring several dimensions of the participant’s behavior or mood. The dimensions, which are expressed as peak scores in Table 2, were rated on a 5-point scale from 0 to 4. Data were the mean of the two monitor ratings at each time-point.

**Table 2.** Peak effects on cardiovascular measures and session monitor ratings of participant behavior and mood assessed throughout the session^+^.

Measure	Low dose	High dose
*Cardiovascular measures (peak effects)*		
Systolic blood pressure (mm Hg)	142.20 (2.45)	155.26 (2.87)***
Diastolic blood pressure (mm Hg)	82.90 (1.35)	89.68 (1.21)***
Heart rate (beats per minute)	78.86 (2.17)	84.06 (2.36)***
*Session monitor ratings (peak effects)* ^a^		
Overall drug effect	1.37 (0.09)	2.90 (0.07)***
Unresponsive to questions	0.13 (0.07)	0.70 (0.12)***
Anxiety or fearfulness	0.50 (0.10)	0.93 (0.15)**
Distance from ordinary reality	0.94 (0.12)	2.68 (0.10)***
Ideas of reference/paranoidthinking	0.05 (0.03)	0.14 (0.05)***
Yawning	0.33 (0.11)	1.28 (0.26)***
Tearing/crying	0.66 (0.14)	2.01 (0.25)***
Nausea/vomiting	0.11 (0.04)	0.44 (0.10)**
Visual effects with eyes open	0.32 (0.09)	1.83 (0.17)***
Visual effects with eyes closed	0.93 (0.09)	1.75 (0.07)***
Spontaneous motor activity	1.12 (0.15)	1.86 (0.30)*
Restless/fidgety	0.83 (0.12)	1.28 (0.15)**
Joy/intense happiness	0.69 (0.12)	1.90 (0.14)***
Peace/harmony	1.08 (0.13)	2.01 (0.13)***
Psychological discomfort	0.34 (0.08)	0.91 (0.15)***
Physical discomfort	0.31 (0.08)	0.62 (0.11)**

+ Data are means (SEM) for peak effects during sessions after low dose (*n*=50) or high dose (*n*=50) psilocybin collapsed across the two dose sequence groups. Asterisks indicate significant differences from the low dose (*p<0.05, **p<0.01, ***p<0.001).

a Maximum possible scores for all monitor ratings were 4 except for visual effects with eyes closed which was 2.

#### Subjective drug effect measures assessed 7 h after psilocybin administration

When psilocybin effects had subsided, participants completed four questionnaires: Hallucinogen Rating Scale (HRS) (Strassman et al., 1994); 5-Dimension Altered States of Consciousness (5D-ASC) (Dittrich, 1998); Mysticism Scale (Experience-specific 9-point scale) (Hood et al., 2001, 2009); and the States of Consciousness Questionnaire (SOCQ) (Griffiths et al., 2006). Thirty items on the SOCQ comprise the Mystical Experience Questionnaire (MEQ30), which was shown sensitive to mystical-type subjective effects of psilocybin in laboratory studies as well as survey studies of recreational use of psilocybin mushrooms (Barrett et al., 2015; MacLean et al., 2012). Four factor scores (Mystical, Positive mood, Transcendence of time and space, and Ineffability) and a mean total score (the mean of all 30 items) were assessed.

#### Therapeutically relevant measures assessed at Baseline, 5 weeks after each session, and 6-month follow-up

Seventeen measures focused on mood states, attitudes, disposition, and behaviors thought to be therapeutically relevant in psychologically distressed cancer patients were assessed at four time-points over the study: immediately after study enrollment (Baseline assessment), about 5 weeks (mean 37 days) after each session (Post-session 1 and 2 assessments), and about 6 months (mean 211 days) after session 2 (6-month follow-up).

The two primary therapeutic outcome measures were the widely used clinician-rated measures of depression, GRID-HAM-D-17 (ISCDD, 2003) and anxiety, HAM-A assessed with the SIGH-A (Shear et al., 2001). For these clinician-rated measures, a clinically significant response was defined as ⩾50% decrease in measure relative to Baseline; symptom remission was defined as ⩾50% decrease in measure relative to Baseline and a score of ⩽7 on the GRID-HAMD or HAM-A (Gao et al., 2014; Matza et al., 2010).

Fifteen secondary measures focused on psychiatric symptoms, moods, and attitudes: BDI, self-rated depression measure (Beck and Steer, 1987); HADS, self-rated separate measures of depression and anxiety, and a total score (Zigmond and Snaith, 1983); STAI, self-rated measure of state and trait anxiety separately (Spielberger, 1983); POMS, Total Mood Disturbance Subscale, self-rated dysphoric mood measure (McNair et al., 1992); BSI, self-rated psychiatric symptoms (Derogatis, 1992); MQOL, self-rated measure of overall quality of life (total score) and meaningful existence (existential subscale) during life-threatening illness (Cohen et al., 1995); LOT-R, self-rated optimism measure associated with illness (Scheier and Carver, 1985); LAP-R Death Acceptance, self-rated scale assessing absence of anxiety about death (Reker, 1992); Death Transcendence Scale, self-rated measure of positive attitudes about death (VandeCreek, 1999); Purpose in Life Test, self-rated measure of life meaningfulness (McIntosh, 1999); and LAP-R Coherence, self-rated scale assessing logically integrated understanding of self, others, and life in general (Reker, 1992).

#### Community observer-rated changes in participant behavior and attitudes assessed at Baseline, 5 weeks after Session 2, and 6-month follow-up

Structured telephone interviews with community observers (e.g. family members, friends, or work colleagues) provided ratings of participant attitudes and behavior reflecting healthy psychosocial functioning (Griffiths et al., 2011). The interviewer provided no information to the rater about the participant or the nature of the research study. The structured interview (Community Observer Questionnaire) consisted of asking the rater to rate the participant’s behavior and attitudes using a 10-point scale (from 1 = not at all, to 10 = extremely) on 13 items reflecting healthy psychosocial functioning: inner peace; patience; good-natured humor/playfulness; mental flexibility; optimism; anxiety (scored negatively); interpersonal perceptiveness and caring; negative expression of anger (scored negatively); compassion/social concern; expression of positive emotions (e.g. joy, love, appreciation); self-confidence; forgiveness of others; and forgiveness of self. On the first rating occasion, which occurred soon after acceptance into the study, raters were instructed to base their ratings on observations of and conversations with the participant over the past 3 months. On two subsequent assessments, raters were told their previous ratings and were instructed to rate the participant based on interactions over the last month (post-session 2 assessment) or since beginning in the study (6-month follow-up). Data from each interview with each rater were calculated as a total score. Changes in each participant’s behavior and attitudes after drug sessions were expressed as a mean change score (i.e. difference score) from the baseline rating across the raters. Of 438 scheduled ratings by community observers, 25 (<6%) were missed due to failure to return calls or to the rater not having contact with the participant over the rating period.

#### Spirituality measures assessed at Baseline, 5 weeks after Session 2, and 6-month follow-up

Three measures of spirituality were assessed at three time-points: Baseline, 5 weeks after session 2, and at the 6-month follow-up: FACIT-Sp, a self-rated measure of the spiritual dimension of quality of life in chronic illness (Peterman et al., 2002) assessed on how the participant felt “on average”; Spiritual-Religious Outcome Scale, a three-item measure used to assess spiritual and religious changes during illness (Pargament et al., 2004); and Faith Maturity Scale, a 12-item scale assessing the degree to which a person’s priorities and perspectives align with “mainline” Protestant traditions (Benson et al., 1993).

#### Persisting effects of the psilocybin session assessed 5 weeks after each session and 6-month follow-up

The Persisting Effects Questionnaire assessed self-rated positive and negative changes in attitudes, moods, behavior, and spiritual experience attributed to the most recent psilocybin session (Griffiths et al., 2006, 2011). At the 6-month follow-up, the questionnaire was completed on the basis of the high-dose session, which was identified as the session in which the participant experienced the most pronounced changes in their ordinary mental processes. Twelve subscales (described in Table 8) were scored.

The questionnaire included three final questions (see Griffiths et al. 2006 for more specific wording): (1) How personally meaningful was the experience? (rated from 1 to 8, with 1 = no more than routine, everyday experiences; 7 = among the five most meaningful experiences of my life; and 8 = the single most meaningful experience of my life). (2) Indicate the degree to which the experience was spiritually significant to you? (rated from 1 to 6, with 1 = not at all; 5 = among the five most spiritually significant experiences of my life; 6 = the single most spiritually significant experience of my life). (3) Do you believe that the experience and your contemplation of that experience have led to change in your current sense of personal well-being or life satisfaction? (rated from +3 = increased very much; +2 = increased moderately; 0 = no change; –3 = decreased very much).

### Statistical analysis

Differences in demographic data between the two dose sequence groups were examined with *t*-tests and chi-square tests with continuous and categorical variables, respectively.

Data analyses were conducted to demonstrate the appropriateness of combining data for the 1 and 3 mg/70 kg doses in the low-dose condition and for including data for the one participant who received 30 mg/70 kg. To determine if the two different psilocybin doses differed in the low-dose condition, *t*-tests were used to compare participants who received 3 mg/70 kg (*n* = 12) with those who received 1 mg/70 kg (*n* = 38) on participant ratings of peak intensity of effect (HRS intensity item completed 7 h after administration) and peak monitor ratings of overall drug effect across the session. Because neither of these were significantly different, data from the 1 and 3 mg/70 kg doses were combined in the low-dose condition for all analyses.

Of the 50 participants who completed the high-dose condition, one received 30 mg/70 kg and 49 received 22 mg/70 kg. To determine if inclusion of the data from the one participant who received 30 mg/70 kg affected conclusions about the most therapeutically relevant outcome measures, the analyses for the 17 measures shown in Tables 4 and 5 were conducted with and without that participant. Because there were few differences in significance (72 of 75 tests remained the same), that participant’s data were included in all the analyses.

To examine acute drug effects from sessions, the drug dose conditions were collapsed across the two dose sequence groups. The appropriateness of this approach was supported by an absence of any significant group effects and any group-by-dose interactions on the cardiovascular measures (peak systolic and diastolic pressures and heart rate) and on several key monitor- and participant-rated measures: peak monitor ratings of drug strength and joy/intense happiness, and end-of-session participant ratings on the Mysticism Scale.

Six participants reported initiating medication treatment with an anxiolytic (2 participants), antidepressant (3), or both (1) between the Post-session 2 and the 6-month follow-up assessments. To determine if inclusion of these participants affected statistical outcomes in the analyses of the 6-month assessment, the analyses summarized in Tables 4, 5, 6, 7 and 8 were conducted with and without these six participants. All statistical outcomes remained identical. Thus, data from these six participants were retained in the data analyses.

For cardiovascular measures and monitor ratings assessed repeatedly during sessions, repeated measures regressions were conducted in SAS PROC MIXED using an AR(1) covariance structure and fixed effects of dose and time. Planned comparison *t*-tests were used to assess differences between the high- and low-dose condition at each time-point.

Peak scores for cardiovascular measures and monitor ratings during sessions were defined as the maximum value from pre-capsule to 6 h post-capsule. These peak scores and the end-of-session ratings (Tables 2 and 3) were analyzed using repeated measures regressions in SAS PROC MIXED with a CS covariance structure and fixed effects of group and dose.

**Table 3.** Participant ratings on questionnaires completed 7 hours after psilocybin administration^+^.

Questionnaire and subscale description	Low dose (post-session)	High dose (post-session)
*Hallucinogen Rating Scale (HRS)*		
Intensity	36.47 (2.78)	63.76 (2.34)***
Somesthesia	15.38 (1.55)	35.62 (2.75)***
Affect	23.79 (2.13)	44.60 (2.54)***
Perception	12.92 (1.76)	41.18 (2.78)***
Cognition	18.88 (2.09)	43.08 (2.54)***
Volition	30.81 (2.02)	37.06 (1.88)*
*5 Dimension Altered States of Consciousness (5D-ASC)*		
Oceanic boundlessness (OBN)	26.86 (3.73)	63.99 (3.78)***
Dread of ego dissolution (DED)	6.89 (1.50)	19.21 (2.38)***
Visionary restructuralization (VRS)	22.41 (2.99)	61.16 (3.48)***
Auditory alterations (AUA)	6.72 (1.87)	14.88 (2.18)***
Vigilance reduction (VIR)	22.74 (2.70)	30.85 (2.24)**
*Mystical Experience Questionnaire (MEQ30)*		
Mystical	24.34 (3.83)	59.58 (4.22)***
Transcendence of time and space	22.38 (2.90)	62.08 (3.38)***
Positive mood	35.84 (4.00)	69.82 (3.82)***
Ineffability	30.80 (4.49)	74.46 (3.67)***
Total	26.90 (3.44)	63.64 (3.56)***
*Mysticism Scale (M scale)*		
Interpretation	48.95 (3.54)	71.45 (2.24)***
Introvertive	44.53 (3.21)	71.20 (2.14)***
Extrovertive	37.48 (3.19)	64.58 (2.81)***
Total	49.36 (3.51)	77.38 (2.40)***

+ All data are expressed as a percentage of maximum possible score. Data are means (1 SEM) for questionnaires completed 7 h after the low-dose (*n* = 50) and high-dose (*n* = 50) sessions collapsed across the two dose sequence groups. Asterisks indicate significant differences from the low dose (*p<0.05, **p<0.01, ***p<0.001).

For the analyses of continuous measures described below, repeated measures regressions were conducted in SAS PROC MIXED using an AR(1) covariance structure and fixed effects of group and time. Planned comparison *t*-tests (specified below) from these analyses are reported. For dichotomous measures, Friedman’s Test was conducted in SPSS for both the overall analysis and planned comparisons as specified below. All results are expressed as unadjusted scores.

For the measures that were assessed in the two dose sequence groups at Baseline, Post-session 1, Post-session 2, and 6 months (Tables 4 and 5), the following planned comparisons most relevant to examining the effects of psilocybin dose were conducted: Between-group comparisons at Baseline, Post 1, and Post 2; and within-group comparisons of Baseline versus Post 1 in both dose sequence groups, and Post 1 versus Post 2 in the Low-Dose-1st (High-Dose-2nd) Group. A planned comparison between Baseline and 6 months collapsed across groups was also conducted. Effects sizes were calculated using Cohen’s *d*.

**Table 4.** Effects of psilocybin on the 11 therapeutically relevant outcome measures assessed at Baseline, Post-session 1 (5 weeks after Session 1), Post-session 2 (5 weeks after Session 2), and 6 months follow-up that fulfilled conservative criteria for demonstrating an effect of psilocybin^+^.

Measure	Group	Assessment time-point			
		Baseline^a^	Post-session 1^b^	Post-session 2^c^	6 months^d^
GRID-HAMD-17 (Depression)	Low-Dose-1st (High-Dose-2nd)	22.32 (0.88)	*14.80 (1.45)*	6.50 (0.86)***	6.95 (1.24)
	High-Dose-1st (Low-Dose-2nd)	22.84 (0.97)	6.64 (1.04)***	6.52 (1.44)	6.23 (1.30)
Beck Depression Inventory (BDI)	Low-Dose-1st (High-Dose-2nd)	18.40 (1.09)	*12.92 (1.58)*	8.17 (1.24)***	8.00 (1.50)
	High-Dose-1st (Low-Dose-2nd)	17.77 (1.61)	7.00 (1.39)**	5.80 (1.41)	6.17 (1.26)
HADS Depression	Low-Dose-1st (High-Dose-2nd)	9.48 (0.71)	*6.04 (0.79)*	4.57 (0.73)*	4.64 (0.72)
	High-Dose-1st (Low-Dose-2nd)	9.81 (0.69)	3.92 (0.74)*	4.28 (0.89)	3.46 (0.66)
HAM-A (Anxiety)	Low-Dose-1st (High-Dose-2nd)	25.68 (0.89)	*16.64 (1.53)*	8.92 (1.14)***	7.95 (1.19)
	High-Dose-1st (Low-Dose-2nd)	25.73 (1.11)	8.48 (1.16)***	7.52 (1.27)	7.04 (1.17)
STAI-Trait Anxiety	Low-Dose-1st (High-Dose-2nd)	47.46 (1.62)	*40.48 (2.11)*	35.48 (2.05)**	36.83 (2.08)
	High-Dose-1st (Low-Dose-2nd)	47.73 (1.91)	34.64 (1.84)*	34.28 (2.25)	35.32 (2.18)
POMS Total Mood Disturbance	Low-Dose-1st (High-Dose-2nd)	51.72 (6.35)	42.48 (7.72)	21.09 (5.81)***	23.50 (6.57)
	High-Dose-1st (Low-Dose-2nd)	56.93 (5.33)	18.96 (5.78)**	17.14 (6.35)	12.52 (5.36)
Brief Symptom Inventory (BSI)	Low-Dose-1st (High-Dose-2nd)	41.76 (4.40)	*33.74 (4.47)*	26.08 (4.53)*	23.50 (3.85)
	High-Dose-1st (Low-Dose-2nd)	40.19 (3.71)	18.08 (3.62)**	16.48 (3.77)	14.35 (3.35)
MQOL (Overall Quality of Life)	Low-Dose-1st (High-Dose-2nd)	5.69 (0.24)	6.17 (0.32)	6.90 (0.34)**	6.88 (0.37)
	High-Dose-1st (Low-Dose-2nd)	5.32 (0.29)	7.14 (0.29)*	7.46 (0.34)	7.65 (0.36)
MQOL (Meaningful Existence)	Low-Dose-1st (High-Dose-2nd)	6.03 (0.30)	6.10 (0.39)	7.30 (0.35)***	7.29 (0.31)
	High-Dose-1st (Low-Dose-2nd)	5.43 (0.29)	7.23 (0.33)*	7.30 (0.38)	7.62 (0.35)
LAP-R Death Acceptance	Low-Dose-1st (High-Dose-2nd)	28.05 (2.04)	29.14 (2.25)	34.95 (1.92)***	34.95 (1.52)
	High-Dose-1st (Low-Dose-2nd)	29.09 (2.07)	36.17 (1.59)*	35.13 (1.90)	36.25 (1.59)
LOT-R (Optimism)	Low-Dose-1st (High-Dose-2nd)	13.56 (0.97)	13.60 (1.23**)**	15.96 (1.12)**	16.68 (1.14)
	High-Dose-1st (Low-Dose-2nd)	14.15 (0.97)	17.23 (0.67)*	17.16 (0.99)	17.43 (0.92)

+ Numerical data show means (SEM) for outcome measures in the two dose sequence groups: (1) those that received a low dose on the 1st session and a high dose on the 2nd (*n* = 25, 25, 24, and 22 at Baseline, Post-session 1, Post-session 2, and 6 months, respectively), and (2) those that received a high dose on 1st session and a low dose on the 2nd (*n* = 26, 25 or 26, 25, and 24 at Baseline, Post-session 1, Post-session 2, and 6 months, respectively). Data are shown for the 11 measures that fulfilled the most conservative criteria for demonstrating psilocybin effects (i.e. showing a significant between-group difference at the Post-session 1 assessment as well as a difference between Post-session 1 and Post-session 2 assessments in the Low-Dose-1st (High-Dose-2nd) Group). Results for the measures not fulfilling these criteria are shown in Table 5.

a In this column (Baseline), there were no significant differences between groups.

b In this column, italic font indicates a within-group significant difference from Baseline (*p*<.05, planned comparison); asterisks indicate significant differences between groups (*p<0.05, **p<0.01, ***p<0.001, planned comparisons); between groups effect size (Cohen’s *d* as absolute values) for the 11 measures from top to bottom were: 1.30, 0.81, 0.56, 1.23, 0.60, 0.70, 0.78, 0.65, 0.65, 0.97, and 0.75.

c In this column, there were no significant differences between groups; asterisks indicate significant differences between the Post-session 1 and Post-session 2 assessments in the Low-Dose-1st (High-Dose-2nd) Group (*p<0.05, **p<0.01, ***p<0.001, planned comparisons); effect size (Cohen’s *d* as absolute values) for the 11 measures from top to bottom were: 1.33, 0.69, 0.40, 1.10, 0.50, 0.64, 0.35, 0.46, 0.66, 0.68, and 0.41.

d The difference between Baseline and 6 months, collapsed across groups, was significant for all 11 measures (*p*<0.001, planned comparison); effect size (Cohen’s *d* as absolute values) for the 11 measures from top to bottom were: 2.98,1.63, 1.65, 3.40, 1.20, 1.26, 1.17, 1.14, 1.12, 0.84, and 0.66.

**Table 5.** Effects of psilocybin on six therapeutically relevant outcome measures assessed at Baseline, Post-session 1 (5 weeks after Session 1), Post-session 2 (5 weeks after Session 2), and 6 months that did not fulfill conservative criteria for demonstrating an effect of psilocybin^+^.

Measure	Group	Assessment time-point			
		Baseline^a^	Post-session 1^b^	Post-session 2^c^	6 months^d^
HADS Total	Low-Dose-1st (High-Dose-2nd)	20.52 (0.92)	*12.04 (1.18)*	9.17 (1.15)*	9.32 (1.22)
	High-Dose-1st (Low-Dose-2nd)	20.88 (0.89)	*9.31 (1.29)*	8.96 (1.53)	8.17 (1.16)
HADS Anxiety	Low-Dose-1st (High-Dose-2nd)	11.04 (0.60)	*6.00 (0.59)*	4.91 (0.60)	4.68 (0.67)
	High-Dose-1st (Low-Dose-2nd)	11.08 (0.53)	*5.38 (0.78)*	4.68 (0.75)	4.71 (0.65)
STAI State Anxiety	Low-Dose-1st (High-Dose-2nd)	42.00 (1.76)	*37.48 (2.49)*	32.83 (2.21)*	32.73 (2.38)
	High-Dose-1st (Low-Dose-2nd)	45.77 (1.98)	*34.36 (2.17)*	31.56 (2.02)	30.25 (1.98)
Death Transcendence Scale	Low-Dose-1st (High-Dose-2nd)	122.12 (4.39)	*127.66 (3.92)*	136.00 (3.62)**	133.36 (3.91)
	High-Dose-1st (Low-Dose-2nd)	117.85 (3.34)	*128.46 (3.99)*	127.25 (4.09)	128.96 (4.07)
Purpose in Life	Low-Dose-1st (High-Dose-2nd)	96.16 (3.32)	*101.80 (3.78)*	106.92 (3.63)*	108.00 (3.36)
	High-Dose-1st (Low-Dose-2nd)	91.04 (3.43)	*106.19 (3.04)*	107.00 (3.73)	108.08 (3.71)
LAP-R Coherence	Low-Dose-1st (High-Dose-2nd)	35.25 (2.36)	*38.14 (2.52)*	43.00 (2.31)*	43.25 (2.09)
	High-Dose-1st (Low-Dose-2nd)	30.86 (1.91)	*36.83 (2.01)*	39.30 (2.05)	40.25 (1.93)

+ Numerical data show means (1 SEM) for primary outcome measures in the two dose sequence groups: (1) those that received a low dose on the 1st session and a high dose on the 2nd (*n* = 25, 25, 24, and 22 at Baseline, Post-session 1, Post-session 2, and 6 months, respectively), and (2) those that received a high dose on 1st session and a low dose on the 2nd (*n* = 26, 26, 25, and 24 at Baseline, Post-session 1, Post-session 2, and 6 months, respectively). Data are shown for the six measures that did not fulfill the most conservative criteria for demonstrating psilocybin effects (i.e. did not show a significant between-group difference at the Post-session 1 assessment as well as a significant difference between Post-session 1 and Post-session 2 assessments in the Low-Dose-1st (High-Dose-2nd) Group).

a In this column, there were no significant differences between groups.

b In this column, italic font indicates a within-group significant difference from Baseline (*p*<0.05, planned comparison); there were no significant between-group differences.

c In this column, there were no significant differences between groups; asterisks indicate significant differences between the Post-session 1 and Post-session 2 assessments in the Low-Dose-1st (High-Dose-2nd) Group (*p<0.05, **p<0.01, planned comparisons); effect size (Cohen’s *d* as absolute values) for the five significant measures (HADS total, STAI State Anxiety, Death Transcendence Scale, Purpose in Life, and LAP-R Coherence, respectively were: 0.51, 0.41, 0.46, 0.28, and 0.49.

d The difference between Baseline and 6 months, collapsed across groups, was significant for all six measures (*p*<0.001, planned comparison); effect size (Cohen’s *d* as absolute values) for the six measures from top to bottom were: 2.34, 2.15, 1.25, 0.58, 0.85, and 0.90.

For measures assessed only at Baseline, Post 2, and 6 months (Table 7), between-group planned comparisons were conducted at Baseline, Post 2, and 6 months. Because measures assessed only at these time-points cannot provide information about the psilocybin dose, data were collapsed across the two dose sequence groups and planned comparisons were conducted comparing Baseline with Post 2 and Baseline with 6 months.

For participant ratings of persisting effects attributed to the session (e.g. Table 8), planned comparisons for continuous and dichotomous measures were conducted between: (1) ratings at 5 weeks after the low versus high-dose sessions; (2) ratings of low dose at 5 weeks versus ratings of high dose at the 6-month follow-up; (3) ratings of high dose at 5 weeks versus ratings of high dose at the 6-month follow-up.

As described above, clinician-rated measures of depression (GRID-HAMD) and anxiety (HAM-A) were analyzed as continuous measures. In addition for both measures, a clinically significant response was defined as ⩾50% decrease in measure relative to Baseline; symptom remission was defined as ⩾50% decrease in measure relative to Baseline and a score of ⩽7. Planned comparisons were conducted via independent *z*-tests of proportions between the two dose sequence groups at Post-session 1, Post-session 2, and 6 months. To determine if effects were sustained at 6 months, planned comparisons were also conducted via dependent *z*-tests of proportions between Post-session 2 versus 6 months in the Low-Dose-1st (High-Dose-2nd) Group, and between Post-session 1 versus 6 months in the High-Dose-1st (Low-Dose-2nd) Group.

Exploratory analyses used Pearson’s correlations to examine the relationship between total scores on the Mystical Experience Questionnaire (MEQ30) assessed at the end of session 1 and enduring effects assessed 5 weeks after session 1. The Post-session 1 measures were ratings on three items from the Persisting Effects Questionnaire (meaningfulness, spiritual significance, and life satisfaction) and 17 therapeutically relevant measures assessed at Baseline and Post 1 (Tables 4 and 5) expressed as difference from baseline scores. Significant relationships were further examined using partial correlations to control for end-of-session participant-rated “Intensity” (item 98 from the HRS). To examine MEQ30 scores as a mediator of the effect of psilocybin dose on therapeutic effects, a bootstrap analysis was done using the PROCESS macro (Hayes, 2013) in SPSS. Bootstrapping is a non-parametric method appropriate for small samples, which was used to estimate 95% confidence intervals for the mediation effect. The PROCESS macro also calculated direct effects on outcome for both group effects and MEQ30.

## Results

### Adverse effects

No serious adverse events attributed to psilocybin administration occurred. A number of adverse events occurred during psilocybin sessions, none of which were deemed to be serious. Except as noted below, all of these adverse events had resolved fully by the end of the sessions. Consistent with previous research (Griffiths et al., 2006, 2011), there were transient moderate increases in systolic and/or diastolic blood pressure after psilocybin. In this study, an episode of elevated systolic blood pressure (>160 mm Hg at one or more time-point) occurred in 34% of participants in the high-dose session and 17% of participants in the low-dose session. An episode of elevated diastolic blood pressure (>100 mm Hg at one or more time-point) occurred in 13% of participants in the high-dose session and 2% of participants in the low-dose session. None of these episodes met criteria for medical intervention. Nausea or vomiting occurred in 15% of participants in the high-dose session and none in the low-dose session. An episode of physical discomfort (any type) occurred in 21% of participants in the high-dose session and 8% in the low-dose session. Also consistent with previous research (Griffiths et al., 2006, 2011), transient episodes of psychological distress during psilocybin sessions (as rated by session monitors) were more common after the high dose than the low dose. Psychological discomfort (any type) occurred in 32% of participants in the high-dose session and 12% in the low-dose session. An episode of anxiety occurred in 26% of participants in the high-dose session and 15% in the low-dose session. One participant had a transient episode of paranoid ideation (2% of high-dose sessions). There were no cases of hallucinogen persisting perception disorder or prolonged psychosis. One participant reported mild headache starting toward the end of the high-dose session and lasting until 9 p.m. that evening. Of the 11 participants for whom headache was assessed on the day after sessions, two reported a delayed moderate headache after the high-dose session.

### Integrity of blinding procedures

After all psilocybin sessions had been completed, the eight study staff members who had served as primary monitors or as assistant monitors for four or more participants completed a questionnaire that asked about their understanding of the experimental design. Although all correctly believed that psilocybin had been administered, five of eight made incorrect inferences about the study design or procedures, including possible administration of three or more dose levels of psilocybin across different participants (four monitors), an inactive placebo (one monitor), other psychoactive compounds such as dextromethorphan (one monitor), or only low psilocybin doses (one monitor).

At the end of each session day, monitors rated their guess of the magnitude of drug dose administered in the capsule that day on a 10 cm line. Although, as expected, the mean (±SE) monitor rating of the dose magnitude of the high psilocybin dose was significantly larger than the low dose (7.0±0.29 vs. 1.7±0.21, *p*<0.001, planned comparison), the distributions of ratings overlapped, with more than 13% of the high-dose sessions being rated as 4 or less and more than 12% of the low-dose sessions being rated as 4 or more. Overall, we conclude that the blinding procedures provided some protection against a priori monitor expectancy strongly determining outcomes of the psilocybin dose manipulation.

### Outcome measures

**Figure 2.**
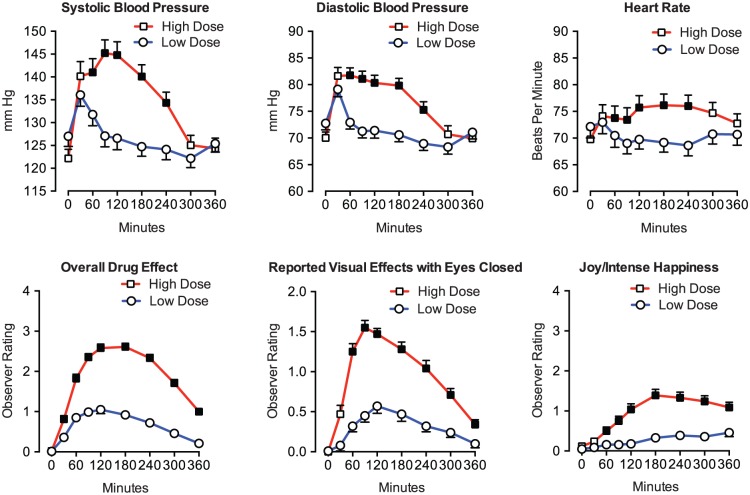
Within-session time-course of psilocybin effects on cardiovascular and observer-rated measures. Cardiovascular (systolic and diastolic blood pressure, and heart rate) and observer (i.e. monitor)-rated overall drug effect, visual effects with eyes closed (as described by the participant), and joy/intense happiness. Data points show means; brackets indicate 1 SEM; circles show data after the low dose (*n* = 50); squares show data after the high dose (*n* = 50). Filled squares indicate the dose conditions were significantly different at the indicated time-point (*p*<0.05, planned comparisons). Y-axes for observer ratings show maximum possible scores.

Psilocybin produced orderly dose- and time-related increases on blood pressure, heart rate, and all 16 monitor-rated dimensions of the participant’s behavior or mood assessed throughout sessions, with a generally similar time-course in both dose conditions (see Figure 2 for illustrative time-course measures). Significant differences between the dose conditions generally first occurred at 30- or 60-min, with the high dose usually showing peak effects from 90–180 min and decreasing toward pre-drug levels over the remainder of the session. Table 2 shows mean peak effects for these measures.

End-of-session measures that assessed subjective experiences during the session were significantly greater after the high than the low dose (Table 3).

**Figure 3.**
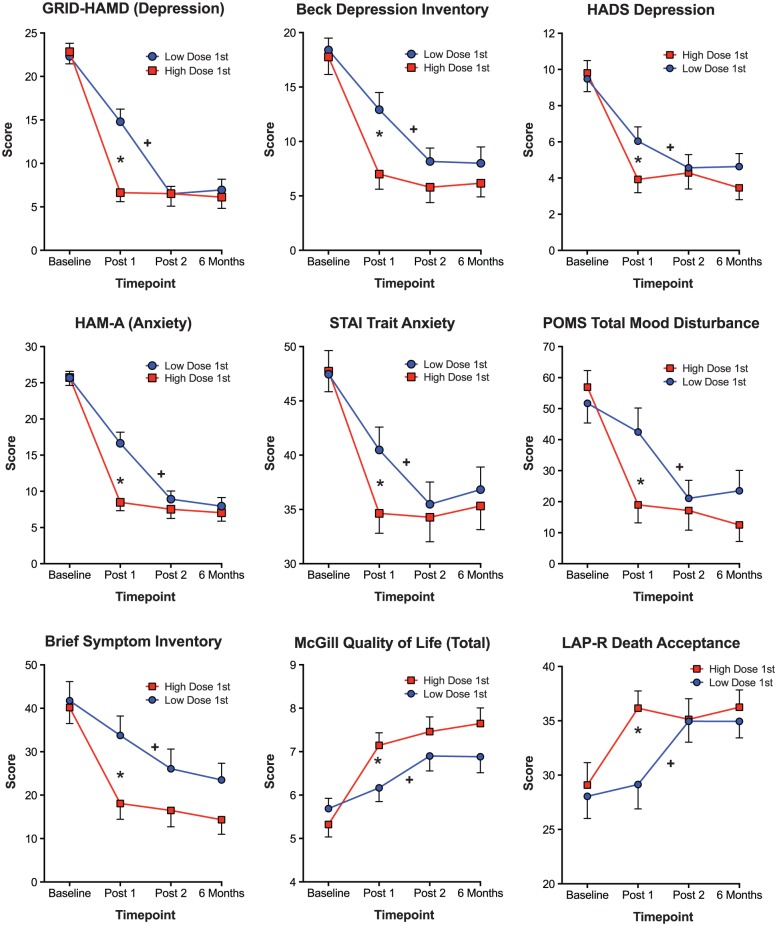
Effects of psilocybin on selected outcome measures that were assessed at Baseline, Post-session 1 (5 weeks after Session 1), Post-session 2 (5 weeks after Session 2), and 6-month follow-up. Data points show means; brackets indicate 1 SEM; circles represent the group that received a low dose on the 1st session and a high dose on the 2nd session (*n* = 25, 25, 24, and 22 at Baseline, Post-session 1, Post-session 2, and 6 months, respectively); squares represent the group that received a high dose on 1st session and a low dose on the 2nd session (*n* = 26, 26, 25, and 24 at Baseline, Post-session 1, Post-session 2, and 6 months, respectively). Star symbol indicates a significant difference between the two groups at the Post-session 1 time-point (*p*<0.05, planned comparison). Cross symbol indicates a significant difference between the Post-session 1 and Post-session 2 time-points in the Low-Dose-1st (High-Dose-2nd) Group (*p*<0.05, planned comparison).

Psilocybin produced large and sustained effects on the two primary clinician-rated therapeutically relevant outcome measures as well as most of the secondary measures assessed at Baseline, 5 weeks after each session, and at 6-month follow-up. Of the 17 measures assessed, 16 showed significant effects (i.e. a between-group difference at the Post-session 1 assessment and/or a difference between Post-session 1 and Post-session 2 assessments in the Low-Dose-1st Group). Conservative criteria for concluding that psilocybin dose affected these outcomes is to consider only those measures that showed both a between-group difference at Post-session 1 and a difference between Post-session 1 and Post-session 2 assessments in the Low-Dose-1st Group. Table 4 shows data for the 11 measures that fulfilled these criteria and Figure 3 shows results graphically for nine of these measures. For the 11 measures, the mean effect size (Cohen’s *d*) for the between-group difference at the Post-session 1 assessment was 0.82, for the within-group difference between Post-session 1 and Post-session 2 in the Low-Dose-1st Group was 0.66, and, for both groups combined, the difference between Baseline and 6 months was 1.55 (see Table 4 footnotes).

Table 5 presents results from six therapeutically relevant outcome measures that did not fulfill conservative criteria for demonstrating an effect of psilocybin. Although none of the measures showed a significant difference between groups at Post-session 1, five of the six showed a significant difference between Post-session 1 and Post-session 2 in the Low-Dose-1st (High-Dose-2nd) Group, and all six measures showed large significant changes in a therapeutically relevant direction (decreases in negative affect and increases in positive attitudes about death and life meaning and coherence) from Baseline to 6-Month Follow-up (mean effect size 1.35).

**Figure 4.**
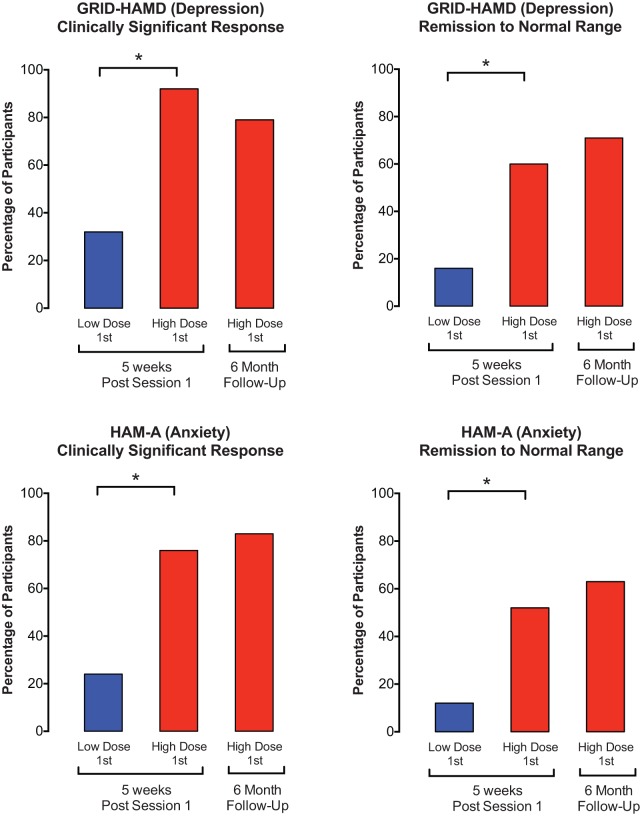
Effects of psilocybin on clinically significant response rate and symptom remission rate as assessed with clinician-rated measures of depression and anxiety. Data are percentage of participants fulfilling criteria at Post-session 1 (5 weeks after Session 1) and at 6 months. Asterisks indicates that the low and high-dose groups were significantly different at 5 weeks (*p*>0.001); data at 6 months show these effects were sustained at follow-up. See Table 6 for other details.

Rates of clinically significant response and symptom remission for the two primary outcome measures of clinician-rated symptoms of depression (GRID-HAMD-17) and anxiety (HAM-A) showed large effects of psilocybin that were sustained at 6 months (Table 6, Figure 4). For instance, 5 weeks after Session 1, 92% of participants in the High-Dose-1st Group showed a clinically significant response (i.e. ⩾50% decrease relative to Baseline) on the GRID-HAMD-17 compared with a 32% response rate in the Low-Dose-1st Group. At 6 months 79% of those in the High-Dose-1st Group continued to show a clinically significant response. Likewise, these percentages for the HAM-A were 76% and 24%, respectively, for the High-Dose 1st Group and Low-Dose-1st Group 5 weeks after Session 1, and 83% for the High-Dose-1st at 6 months. An analogous pattern of results was shown for symptom remission to normal range (i.e. ⩾50% decrease relative to Baseline and a score of ⩽7 on GRID-HAMD-17 or HAM-A), with rates of symptom remission of 60% and 52% for depression and anxiety, respectively, 5 weeks after the high psilocybin dose in Session 1, and with rates of 71% and 63%, respectively, sustained at 6 months. Collapsing across the two dose sequence groups, the overall rate of clinical response at 6 months was 78% and 83% for depression and anxiety, respectively, and the overall rate of symptom remission at 6 months for all participants was 65% and 57%, respectively.

**Table 6.** Percentage of participants with clinically significant response rate and symptom remission rate as assessed with the clinician-rated measures of depression and anxiety^+a^.

Measure	Group	Assessment time-point					
		Post-session 1		Post-session 2		6 months^b^	
		Clinical response	Symptom remission	Clinical response	Symptom remission	Clinical response	Symptom remission
GRID-HAMD-17 (Depression)	Low-Dose-1st (High-Dose-2nd)	32%	16%	75%	58%	77%	59%
	High-Dose-1st (Low-Dose-2nd)	92%***	60%**	84%	68%	79%	71%
HAM-A (Anxiety)	Low-Dose-1st (High-Dose-2nd)	24%	12%	83%	42%	82%	50%
	High-Dose-1st (Low-Dose-2nd)	76%***	52%**	80%	60%	83%	63%

+ Data are percentage of participants fulfilling criteria at Post-session 1 (5 weeks after Session 1), Post-session 2 (5 weeks after Session 2), and 6 months. Clinical response was defined as ⩾50% decrease in measure relative to Baseline; Symptom remission was defined as ⩾50% decrease in measure relative to Baseline and a score of ⩽7 on GRID-HAMD-17 or HAM-A. For the Post-session 1, Post-session 2, and 6-month time-points, respectively, the number of participants was 25, 24, and 22 in the Low-Dose-1st (High-Dose-2nd) Group, and 25, 25, and 24 in the High-Dose-1st (Low-Dose-2nd) Group.

a Within each data column, asterisks indicate significant differences between groups (*p<0.05, **p<0.01, ***p<0.001, planned comparisons, *z*-tests).

b Effects of psilocybin on response and remission were sustained at 6 months as indicated by an absence of significant difference (*p*>0.05, planned comparisons, *z*-tests) between (1) Post-session 2 vs. 6 months in the Low-Dose-1st (High-Dose-2nd) Group and (2) Post-session 1 vs. 6 months in the High-Dose-1st (Low-Dose-2nd) Group. Overall response and remission rates were somewhat higher at 6 months when data were excluded for the six participants who initiated treatment with an antidepressant or anxiolytic between Post-session 2 and 6 months: on the GRID-HAMD-17 mean response and remission rate across the two dose sequence groups at 6 months increased from 78% to 83% and from 65% to 68%, respectively. On the HAM-A these rates increased from 83% to 85% and from 57% to 60%, respectively.

Community observer ratings showed significant positive changes in participants’ attitudes and behavior at the two post-psilocybin assessment time-points (Table 7). All three measures of spirituality showed similar increases (Table 7). As with the measures shown in Table 4, these measures show significant changes in the expected directions at Post-session 2 that were generally sustained at the 6-month follow-up.

**Table 7.** Community observer ratings of participant attitudes and behavior, and three measures of spirituality assessed at Baseline, Post-session 2 (5 weeks after Session 2), and 6 months, collapsed across the two drug sequence groups*.

Measure	Assessment time-point		
	Baseline	Post-session 2^a^	6 months^b^
*Community observer ratings of positive changes in attitudes & behavior*			
Total score	81.62 (1.61)	93.79 (1.70)***	94.41 (1.66)***
*FACIT-Sp – Spiritual well-being in chronic illness*			
Total score (% of maximum score)	44.92 (2.71)	68.13 (3.62)***	70.79 (3.17)***
*Faith Maturity Scale*			
Total score (% of maximum score)	49.73 (2.71)	53.94 (3.39)*	55.56 (3.29)*
*Spiritual/Religious Outcome Scale*			
Total score (% maximum score)	48.53 (3.97)	64.67 (3.54)***	63.41 (3.80)***

* Numerical data show means (1 SEM) for outcome measures collapsed across the two dose sequence groups (*n* = 51, 50, and 46 at Baseline, Post-session 2, and 6 months, respectively). The two dose sequence groups were not significantly different from each other at Baseline, Post-session 2, and 6-month assessments (planned comparisons). Asterisks indicate significant differences from Baseline (*p<0.05, **p<0.01, ***p<0.001, planned comparisons).

a In this column, effect size (Cohen’s *d* as absolute values) for the four measures from top to bottom were: 1.06, 1.03, 0.20, 0.61.

b In this column, effect size (Cohen’s *d* as absolute values) for the four measures from top to bottom were: 1.14, 1.28, 0.28, and 0.55.

Table 8 shows participant ratings of persisting effects attributed to the session experiences rated 5 weeks after the low- and high-dose psilocybin sessions, and, again, for the high-dose session at the 6-month follow-up. The high dose produced significantly greater ratings of positive persisting effects on attitudes about life and self, mood changes, social effects, behavior, and spirituality. These effects were sustained at 6-month follow-up. Negative ratings of these dimensions were low and not significantly different between conditions. The high-dose experiences were rated as producing significantly greater personal meaning, spiritual significance and increased well-being or life satisfaction, with differences sustained at 6 months.

**Table 8.** Participant ratings of persisting effects attributed to the session on ratings completed 5 weeks after the low-dose and high-dose psilocybin sessions, and, again, retrospectively for the high-dose session 6 months after the second session^+^.

Questionnaire and subscale description	Assessment time-point		
	Low dose (5 weeks)	High dose (5 weeks)	High dose 6-month follow-up
*Persisting Effects Questionnaire* (% of maximum score)			
Positive attitudes about life	39.57 (3.91)	57.78 (3.10)***	61.17 (3.51)***
Negative attitudes about life	3.82 (0.99)	5.08 (1.54)	3.18 (0.96)
Positive attitudes about self	35.16 (3.80)	50.70 (3.46)***	54.78 (3.37)***
Negative attitudes about self	3.89 (0.86)	4.80 (1.43)	3.52 (1.16)
Positive mood changes	36.85 (3.99)	49.06 (3.45)***	55.32 (3.58)***
Negative mood changes	3.42 (1.18)	5.42 (1.57)	3.00 (1.18)
Altruistic/positive social effects	35.60 (3.79)	47.42 (3.49)***	51.11 (3.69)***
Antisocial/negative social effects	3.55 (1.11)	3.73 (1.06)	2.51 (0.90)
Positive behavior changes	48.40 (4.66)	59.60 (4.02)***	64.78 (4.03)***
Negative behavior changes	1.60 (1.27)	3.60 (1.97)	0.87 (0.61)
Increased spirituality	37.07 (4.31)	52.48 (3.88)***	57.43 (4.17)***
Decreased spirituality	1.68 (0.63)	1.88 (0.68)	1.27 (0.39)
*How personally meaningful was the experience? (maximum score=8)*	4.62 (0.31)	6.38 (0.20)***	6.65 (0.18)***
Top 5 most meaningful of life, including single most (% of participants)	24%	62%***	67.4%***
*How spiritually significant was the experience? (maximum score=6)*	3.16 (0.24)	4.46 (0.19)***	4.78 (0.17)***
Top 5 most spiritually significant of life, including single most (% of participants)	24%	66%***	69.6%***
*Did the experience change your sense of well-being or life satisfaction? (maximum score=3)*	1.50 (0.19)	2.20 (0.16)***	2.33 (0.14)***
Increased well-being or life satisfaction moderately or very much (% of participants)	52%	86%***	82.6%***

+ Except where noted, numerical data show means (1 SEM) for persisting effects ratings 5 weeks after the low-dose session (*n* = 50), 5 weeks after the high-dose session (*n* = 50), and, again, retrospectively for the high-dose session 6 months after the second session (*n* = 46). There were no significant differences between ratings of the high dose at 5 weeks after the session vs. the 6-month follow-up. Asterisks indicate significant differences from ratings obtained 5 weeks after the low dose session (*p<0.05, **p<0.01, ***p<0.001, planned comparisons).

**Figure 5.**
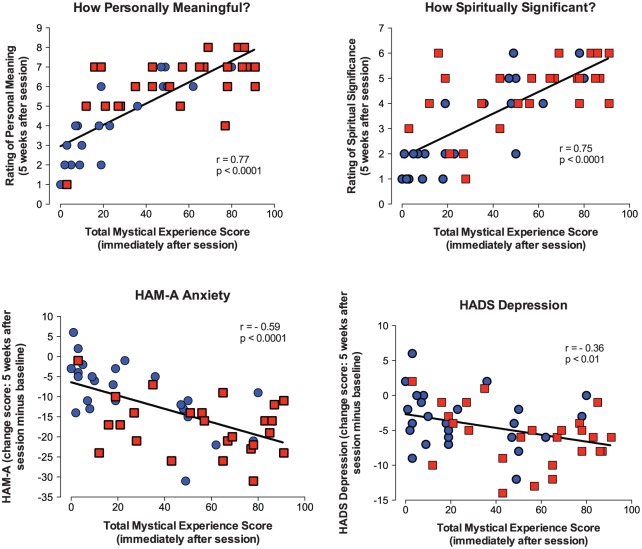
Relationship between the Mystical Experience Questionnaire (MEQ30) total score assessed at end of Session 1 and several illustrative outcome measures assessed 5 weeks after Session 1. Each panel shows scores on an outcome measure assessed 5 weeks after Session 1 as a function of the total MEQ30 score obtained 7 h after psilocybin administration on Session 1. MEQ30 scores are expressed as a percentage of maximum possible score. Data points represent individual participants (*n* = 50 or 51); blue circles represent the group that received the low dose on the 1st session; red squares represent the group that received the high dose on the 1st session. Correlation coefficients and *p*-values are shown.

Mystical experience scores (MEQ30) assessed at the end of Session 1 correlated significantly with 18 of 20 measures assessed 5 weeks after the session: ratings of meaningfulness (*r* = 0.77), spiritual significance (*r* = 0.75), increased life satisfaction (*r* = 0.53), GRID-HAMD (*r* = −0.41), BDI (*r* = −0.30), HADS Depression (*r* = −0.36), HADS Total (*r* = −0.41), HADS Anxiety (*r* = −0.34), HAM-A (*r* = −0.59), STAI-Trait Anxiety (*r* = −0.31), POMS Total Mood Disturbance (*r* = −0.35) BSI (*r* = −0.38), MQOL (*r* = 0.32), MQOF-meaningful existence (*r* = 0.41), LAP-R Death Acceptance (*r* = 0.38), Death Transcendence Scale (*r* = 0.31), Purpose in Life (*r* = 0.29), LAP-R Coherence (*r* = 0.41). Figure 5 shows some of these effects. To further examine the contribution of mystical experience to these outcome measures, partial correlations were conducted to control for the participant-rated intensity of drug effect, which, like mystical experience, was assessed at the end of the session. This analysis continued to show significant effects of mystical experience on 11 of these 18 measures (meaningfulness, spiritual significance, life satisfaction, GRID-HAMD, HADS Depression, HADS Total, HADS Anxiety, HAM-A, BSI, MQOL-meaningful existence and LAP-R Coherence). Finally, a mediation analysis showed that MEQ30 score was a significant mediator of the effect of psilocybin dose on seven of these outcome measures. Point estimates and bias-corrected 95% confidence intervals for the indirect effects of the mediation analysis were: meaningfulness (1.43 [0.72–2.44]), spiritual significance (1.19 [0.59–2.10]), life satisfaction (0.60 [0.218–1.19]), HADS Anxiety (−1.50 [−3.50 to −0.33]), HADS Depression (−1.11 [−2.79 to −0.02]), HADS Total (−2.62 [−5.74 to −0.72]), and HAM-A (−3.93 [−7.88 to −1.52]).

## Discussion

The present study demonstrated the efficacy of a high dose of psilocybin administered under supportive conditions to decrease symptoms of depressed mood and anxiety, and to increase quality of life in patients with a life-threatening cancer diagnosis. Eleven of 17 therapeutically relevant measures fulfilled conservative criteria for demonstrating efficacy of the high dose of psilocybin (Table 4, Figure 3). The data show that psilocybin produced large and significant decreases in clinician-rated and self-rated measures of depression, anxiety or mood disturbance, and increases in measures of quality of life, life meaning, death acceptance, and optimism. These effects were sustained at 6 months. For the clinician-rated measures of depression and anxiety, respectively, the overall rate of clinical response at 6 months was 78% and 83% and the overall rate of symptom remission was 65% and 57%. Participants attributed to the high-dose experience positive changes in attitudes about life, self, mood, relationships and spirituality, with over 80% endorsing moderately or higher increased well-being or life satisfaction. These positive effects were reflected in significant corresponding changes in ratings by community observers (friends, family, work colleagues) of participant attitudes and behavior.

The results substantially extend the findings of a recent double-blind pilot study with a lower dose of psilocybin (14 mg/70 kg) in cancer patients that showed non-significant trends for benefits of psilocybin compared with placebo (niacin) on measures of depression and anxiety, with some significant decreases relative to baseline demonstrated at 1 to 6 months (Grob et al., 2011).

The time-course, magnitude, and qualitative features of the high dose of psilocybin on session days were consistent with those observed in previous studies in healthy volunteers (Griffiths et al., 2006, 2011; Johnson et al., 2012).

The significant association of mystical-type experience (MEQ30) during Session 1 with most of the enduring changes in therapeutic outcome measures 5 weeks later (Figure 5) is consistent with previous findings showing that such experiences on session days predict long-term positive changes in attitudes, mood, behavior, and spirituality (Garcia-Romeu et al., 2014; Griffiths et al., 2008, 2011). For most measures, this relationship continued to be significant when the intensity of overall psilocybin effect was controlled in a partial correlation analysis. This suggests that mystical-type experience per se has an important role apart from overall intensity of drug effect. Finally, a mediation analysis further suggested that mystical-type experience has a mediating role in positive therapeutic response.

The observed decreases in psychological distress and anxiety about death may relate to recent epidemiological findings that lifetime psilocybin use was associated with significantly reduced odds of past month psychological distress and suicidality (Hendricks et al., 2015).

An innovative feature of the study design was that participants and staff monitors were given instructions that obscured the actual psilocybin dose conditions to facilitate blinding and minimize expectancy effects, which are believed to be a significant determinant of classic hallucinogen effects (Griffiths et al., 2006; Metzner et al., 1965). Evidence of some success of this blinding was provided in a post-study questionnaire completed by staff and by significant treatment effects observed after Session 1 in participants who received the very low dose of psilocybin. Although it was assumed that 1 mg/70 kg would be largely pharmacologically inactive, some pharmacological activity of this dose cannot be ruled out entirely. Thus, it might have been preferable to use an even lower dose of psilocybin (e.g. 0.01 mg/70 kg) to assure pharmacological inactivity while maintaining the benefit of the instruction that psilocybin would be administered on each session. Although the low-dose comparison condition and instructions to participants and staff facilitated blinding and minimized expectancy effects, it should be noted that these experimental design features may be difficult to implement in research settings that require complete disclosure of specific study conditions or arms.

Several additional experimental limitations should be noted. Participants were crossed over to the alternative dose condition after 5 weeks. Although this allowed assessment of acute and persisting effects of psilocybin in all study participants, it precluded double-blind assessment of efficacy of the high dose of psilocybin based on across group comparisons after 5 weeks. As in previous research, the study documented enduring increases in positive changes in attitudes and mood on both the participant-rated Persisting Effects Questionnaire and on the Community Observer Questionnaire (Griffiths et al., 2006, 2011). However, neither of these measures has been independently validated. Likewise, although the finding of significant decreases in depression and anxiety symptoms on both participant-rated and clinician-rated measures is a strength, the inclusion of blinded clinician ratings would further strengthen the study. The relatively small sample (*n* = 51) that was highly educated and predominately White limits the generality of conclusions.

Finally, it is important to note that the overall approach of treating cancer-related psychological distress with psilocybin is limited by a variety of exclusion criteria (see online Supplementary material) and by the significant time and cost of professional support provided before, during, and after the psilocybin session. Patients may also be reluctant to participate in such an intervention because high doses of psilocybin have sometimes been associated with transient episodes of psychological distress or anxiety in patients (current study and studies in healthy volunteers, Griffiths et al., 2006, 2011).

The neuropsychopharmacological mechanisms of psilocybin therapeutic effects remain speculative (Carhart-Harris et al., 2012, 2014; Nichols, 2016; Vollenweider and Kometer, 2010). As a 5-HT_2A_ agonist, the psilocybin metabolite psilocin directly and indirectly affects various brain cortical and subcortical areas and alters brain network dynamics (Carhart-Harris et al., 2012, 2014; Vollenweider and Kometer, 2010). Precisely how the enduring therapeutically relevant psilocybin effects are reflected in long-term alteration of cortical networks or other neuroplastic changes remains to be established.

## Conclusions

When administered under psychologically supportive, double-blind conditions, a single dose of psilocybin produced substantial and enduring decreases in depressed mood and anxiety along with increases in quality of life and decreases in death anxiety in patients with a life-threatening cancer diagnosis. Ratings by patients themselves, clinicians, and community observers suggested these effects endured at least 6 months. The overall rate of clinical response at 6 months on clinician-rated depression and anxiety was 78% and 83%, respectively. A multisite study in a larger and more diverse patient population should be conducted to establish the generality and safety of psilocybin treatment of psychological distress associated with life-threatening cancer.

## Supplementary Material

Supplementary material
