# Supplementary material for: Psilocybin produces substantial and sustained decreases in depression and anxiety in patients with life-threatening cancer: A randomized double-blind trial
Source: J Psychopharmacol. 2016 Nov 30;30(12):1181–97. doi: 10.1177/0269881116675513 (PMC5367557; doi:10.1177/0269881116675513)
Supplement: Supplementary material [file JOP675513_Supplementary_material-revised.pdf]

Supplemental Content

Griffiths, R.R., Johnson, M.W., Carducci, M.A., Umbricht, A., Richards, W.A. Richards, B.D., Cosimano, M.P. and Klinedinst, M.A. Psilocybin produces substantial and sustained decreases in depression and anxiety in patients with life-threatening cancer: A randomized double-blind trial

This supplementary material has been provided by the authors to give readers additional information about the study.

Detailed inclusion and exclusion criteria

Inclusion criteria

- 21 to 80 years old
- Have a high school or equivalent (e.g. GED) level of education. Volunteers without a high school or equivalent education must demonstrate reading literacy and comprehension sufficient for understanding the consent form and study questionnaires, as evaluated by study staff obtaining consent.
- Has or has had a cancer diagnosis that is potentially life-threatening. Patients with an active cancer (e.g. stage III or IV with a poor prognosis) or disease progression or recurrence are eligible. Patients who do not have an active cancer or disease progression or disease recurrence are only eligible if at least 1 year has elapsed since their diagnosis.
- Have an ECOG performance status of 0, 1, or 2.
- Have a DSM-IV psychiatric diagnosis, as determined by the SCID, of one or more of the following Axis I psychiatric disorders that is judged to have been precipitated by or exacerbated by the psychological stress of the cancer diagnosis: Generalized Anxiety Disorder; Acute Stress Disorder; Posttraumatic Stress Disorder; Major Depressive Disorder (mild or moderate severity); Dysthymic Disorder; Adjustment Disorder with Anxiety; Adjustment Disorder with Depressed Mood; Adjustment Disorder with Mixed Anxiety and Depressed Mood; Adjustment Disorder with Disturbance of Conduct; Adjustment Disorder with Disturbance of Emotions and Conduct. Psychiatric diagnosis are determined by Johns Hopkins staff.
- Patients receiving chemotherapy, hormonal therapy, radiation therapy, biologic therapies may participate while receiving those therapies. Continuing hormonal therapy, chemotherapy, or radiation treatment is acceptable if the patient is tolerating the therapy or treatment in a sufficient fashion to allow administration of oral psilocybin.
- Agree that for one week preceding each psilocybin session, he/she will refrain from taking any nonprescription medication, nutritional supplement, or herbal supplement except when approved by the research team. Exceptions will be evaluated by the research team and will include acetaminophen, non-steroidal anti-inflammatory drugs, and common doses of vitamins and minerals.
- Agree not to use nicotine for at least 2 hours before psilocybin administration, and not again until questionnaires have been completed approximately 7 hours after psilocybin administration.
- Agree to consume approximately the same amount of caffeine-containing beverage (e.g., coffee, tea) that he/she consumes on a usual morning, before arriving at the research unit on the mornings of psilocybin session days. If the patient does not routinely consume caffeinated beverages, he or she must agree not to do so on psilocybin session days.

- Agree not to take any PRN medications on the mornings of psilocybin sessions, with the exception of daily opioid pain medication. Non-routine PRN medications for treating breakthrough pain that were taken in the 24 hours before the psilocybin session may result in rescheduling the treatment session, with the decision at the discretion of the investigators.
- Agree to refrain from using any psychoactive drugs, including alcoholic beverages, within 24 hours of each psilocybin administration. As described elsewhere, exceptions include daily use of caffeine, nicotine, and opioid pain medication.

Exclusion criteria

General Medical Exclusion Criteria

- Cancer with known CNS involvement, or other major CNS disease. In addition to diagnostic results provided by the referring physician, patients will undergo a neurological exam at the study site. Any patient with evidence of a focal deficit will be excluded.
- Patients will be excluded if they are in treatment in another clinical trial involving an investigational product for treatment of cancer.
- Hepatic dysfunction as indicated by the following values:
  -- GGT > 3 x ULN (upper limit of norm)
  -- AST > 3 x ULN
  -- ALT > 3 x ULN
  -- Tot Bili > 3.0 mg/dl
- Known paraneoplastic syndrome or "ectopic" hormone production by the primary tumor such as the patient could have or be at risk for hypercalcemia, Cushing's syndrome, hypoglycemia, syndrome of inappropriate antidiuretic hormone secretion, or carcinoid syndrome
- Cardiovascular conditions: uncontrolled hypertension, angina, a clinically significant ECG abnormality (e.g. atrial fibrilation), TIA in the last 6 months, stroke, peripheral or pulmonary vascular disease (no active claudication)
- Blood pressure exceeding 140 systolic or 90 diastolic
- Epilepsy with history of seizures
- Renal insufficiency (creatinine clearance < 40 ml/min using the Cockraft and Gault equation)
- Insulin-dependent diabetes; if taking oral hypoglycemic agent, then no history of hypoglycemia
- Females who are pregnant (positive pregnancy test) or nursing, or are not practicing an effective means of birth control
- Currently taking on a regular (e.g., daily) basis: investigational agents, psychoactive prescription medications (e.g., benzodiazepines), medications having a primary pharmacological effect on serotonin neurons (e.g., odansetron), or medications that are MAO inhibitors. Long-acting opioid pain medications (e.g. oxycodone sustained release, morphine sustained release -- which are usually taken at 12 hour intervals) will be allowed if the last dose occurred at least 6 hours before psilocybin administration; such medication will not be taken again until at least 6 hours after psilocybin administration.
- For individuals who have intermittent or PRN use of investigational agents, psychoactive prescription medications, medications having a primary pharmacological effect on serotonin neurons, or medications that are MAO inhibitors, psilocybin sessions will not be conducted until at least 5 half-lives of the agent have elapsed after the last dose.
- Patients will be excluded if they are currently using any the following of potent metabolic inducers or inhibitors: Inducers - Rifamycin (rifampin, rifabutin, rifapentine), anticonvulsants (carbamazepine, phenytoin, phenobarbital), nevirapine, efavirenz, Taxol, dexamethasone), St Johns Wort; Inhibitors - all HIV protease inhibitors, itraconazole, ketoconazole, erythromycin, clarithromycin, troleandomycin.

- Patients will be excluded if it is a medical requirement that they receive any of the following drugs with low therapeutic index within 12 hours after receiving psilocybin: ergot alkaloids, pimozide, midazolam, triazolam, lovastatin, simvastatin, fentanyl.

Psychiatric Exclusion Criteria

- Individuals with severity of depression or anxiety symptoms warranting immediate treatment with antidepressant or daily anxiolytic medication (e.g., due to suicidal ideation). Patients will be interviewed to determine if referral (e.g., to Community Psychiatry) is necessary. For individuals who are consented and screened, we will notify the referring physician as to: 1) whether the individual enrolled in the study or not, and 2) if disqualified, why the individual was disqualified. If disqualification was based on severe depression or anxiety (e.g., suicidal ideation), this will be included in the information conveyed to the referring physician. Permission for this contact will be obtained from the participant.
- Current or past history of meeting DSM-IV criteria for Schizophrenia, Psychotic Disorder (unless substance-induced or due to a medical condition), or Bipolar I or II Disorder
- Current or past history within the last 5 year of meeting DSM-IV criteria for alcohol or drug dependence (excluding caffeine and nicotine).
- Have a first or second degree relative with schizophrenia, psychotic disorder (unless substance induced or due to a medical condition), or bipolar I or II disorder.
- Currently meets DSM-IV criteria for Dissociative Disorder, Anorexia Nervosa, Bulimia Nervosa, or other psychiatric conditions judged to be incompatible with establishment of rapport or safe exposure to psilocybin.

Supplemental Information on Study Location, Dates, and Participant Completion

The study was conducted at the Behavioral Biology Research Center at Johns Hopkins Bayview Medical Center. Enrollment began October 2007 and final follow-up data were obtained in November 2014. As specified in the protocol, enrollment was stopped when 44 volunteers completed all assessments including the 6-month follow-up. At that time, volunteers who were enrolled but who had not completed all assessments were continued in the study until completion or drop-out. This procedure resulted in a total of 46 volunteers who completed the-6 month follow-up.

Supplemental Information on Randomization and Blinding

Volunteers who passed screening were accepted into the study and were sequentially assigned according to the randomization schedule to one of two drug sequence conditions (Low-Dose-1st Group and High-Dose-1st Group). The randomization was managed by the Research Pharmacy and investigators, research staff, and participants were blinded to the randomization sequence. The randomization schedule was generated with a random number table with the constraint that the same condition never occur more than three times consecutively.

Supplemental Information about Power Calculations

Although the primary interest in conducting the study was to determine the effects of the psilocybin intervention on measures of depressed mood and anxitey, statistical power calculations were based on a specific outcome measure (Pahnke Richards Mystical Experience Questionnaire, PRMEQ) for which we had substantial psilocybin data. In the manuscript, we report large effects of psilocybin on the MEQ30, which is a psychometrically-improved version of the PRMEQ comprised of a subset of items from the PRMEQ, Barrett et al., Journal of Psychopharmacology, 2015, 29(11), 1182-1190. Before administration of the first dose of psilocybin, we added our primary therapeutically-relevant outcome measures for depressed mood (GRID-HAM-D-17, GRID Hamilton Rating Scale for

Depression), and anxiety, (HAM- A, Hamilton Anxiety Rating Scale) assessed with the SIGH-A. These clinician rated measures are generally considered "gold standard" assessments of depression and anxiety. Relevant data were not available for estimating statistical power of psilocybin on these specific measures. We also added a range of other secondary therapeutically-relevant measures at the same time, including the HADS, FACIT, Purpose in Life, and Community Observer Ratings. We did not believe the addition of the HAM-D, HAM-A or other measures warranted revising the statistical plan because the statistical plan remained scientifically well-justified.

Supplemental Information about Deaths of Study Participants

No serious adverse events occurred that were judged to be attributable to psilocybin administration. Of the 56 participants who were randomized (Figure 1 in the publication), 3 died due to progression of their cancer before the 6-month follow-up. To our knowledge, an additional 9 participants died due to progression of their cancer between the 6-month follow-up and November 2016. There was a death by suicide of a study participant 11 days after the first session, which involved the administration of the placebo-like very low dose of psilocybin (1 mg/70 kg). This volunteer reported feeling bored and was discontinued from the study after insisting upon leaving this study session early. There was no behavioral impairment and no adverse sequelae on follow-up later that day and over the subsequent several days. Our conclusion, which was reported to the Johns Hopkins IRB and to the FDA, was that the suicide was not related to the research procedures or to psilocybin.
